# Clinical and Genotypic Features of Norovirus Enteritis in Children Complicated by Convulsions: A Retrospective Study in Kunming, China

**DOI:** 10.1155/cjid/9940384

**Published:** 2026-01-09

**Authors:** Suqi Xu, Mingying Wang, Canchun Zhao, Jiahui Fang, Shumei He, Zhuoheng Li, Haoyu Ma, Qiangming Sun, Hongchao Jiang

**Affiliations:** ^1^ Department of Gastroenterology, Kunming Children’s Hospital (Affiliated Children’s Hospital of Kunming Medical University), Kunming, 650228, China; ^2^ Department of Traditional Chinese Medicine, Yunnan University of Chinese Medicine, Kunming, 650500, China, ynutcm.edu.cn; ^3^ Department of Pediatrics, Kunming Children’s Hospital (Affiliated Children’s Hospital of Kunming Medical University), Kunming, 650228, China; ^4^ Institute of Medical Biology, Chinese Academy of Medical Sciences, Kunming, 650118, China, cacms.ac.cn; ^5^ Department of Clinical Laboratory, Kunming Children’s Hospital (Affiliated Children’s Hospital of Kunming Medical University), Kunming, 650228, China

**Keywords:** convulsion, genotype, norovirus enteritis, VP1

## Abstract

**Purpose:**

Although studies have described the clinical characteristics of norovirus enteritis (NoVE) associated with convulsions, research on its molecular epidemiology remains limited. Therefore, this study aimed to investigate both the clinical manifestations and genotypic features of NoVE complicated by convulsions.

**Methods:**

NoVE children admitted to our hospital between November 2021 and May 2022 were divided into NoVE complicated with convulsion group and NoVE group. Then, we screened the risk factors of NoVE complicated with convulsion based on multivariate regression analysis. The stool samples were collected, followed by amplification of VP1 sequences of GI and GII and phylogenetic analysis of VP1 in both groups.

**Results:**

Logistic regression analysis showed that elevated serum CK‐MB levels and fever were independent risk factors for convulsions in NoVE patients, while a lower frequency of diarrhea was an independent protective factor. GII.3 genotype was merely detected in 12 NoVE samples (14.81%) and was detected in 5 (15.19%) NoVE with convulsion samples. The GII.4 genotype was detected in 28 (84.85%) samples of the NoVE with convulsion group and 69 (85.19%) samples from the NoVE group.

**Conclusions:**

Elevated serum CK‐MB concentration, fever, and frequency of diarrhea were independent influencing factors for NoVE complicated by convulsions.

## 1. Introduction

Norovirus (NoV), a highly contagious virus, is responsible for norovirus enteritis (NoVE) worldwide, particularly in the children under 2 years of age [1]. Typical clinical features of NoVE include diarrhea, vomiting, and abdominal pain. In some cases, the disease may progress to more severe manifestations, posing a significant public health challenge [2]. The NoV genome contains three open reading frames (ORFs) encoding a nonstructural polyprotein, the major capsid gene (VP1), and the minor capsid gene (VP2), with VP1 exhibiting high genetic variability [3]. It is classified into at least 10 genogroups, among which GI and GII are primarily responsible for human infections [4]. Specifically, GII genotype, particularly GII.4, accounts for the majority of outbreaks and sporadic cases worldwide [5].

While NoVE is generally self‐limiting, some children develop convulsions [6]. These convulsive episodes may lead to central nervous system (CNS) injury or even epilepsy in children, resulting in long‐term neurological sequelae. Despite this clinical significance, the pathogenesis of NoVE complicated by convulsions remains poorly understood [7]. Previous research studies have largely focused on describing clinical characteristics, whereas studies examining molecular epidemiology and genotype‐specific associations with convulsions are limited [8–11]. Furthermore, there are currently no approved drugs or vaccines targeting NoV infection complicated by convulsions [12].

These gaps highlight the need for a systematic investigation of both the clinical and molecular features of NoVE associated with convulsions. Specifically, the mechanisms remain unclear, including which clinical factors increase the risk of convulsions, how different NoV genotypes contribute to disease severity, and what evolutionary characteristics underlie their pathogenicity. Therefore, this study was designed to identify clinical risk factors associated with convulsions in children with NoVE and to characterize the genetic diversity of NoV strains as well as their associations with clinical manifestations through phylogenetic analysis.

## 2. Materials and Methods

### 2.1. Part I

#### 2.1.1. Subjects

We retrospectively included children diagnosed with NoVE who were admitted to Kunming Children’s Hospital between November 2021 and May 2022. NoV infection was detected using real‐time PCR, consistent with the diagnostic approach recommended by the U.S. CDC for outbreaks and surveillance [13]. The inclusion criteria were as follows: (a) children younger than 5 years with a disease duration of less than 2 weeks; (b) laboratory‐confirmed NoV infection based on stool samples detected by real‐time PCR; (c) absence of coinfection with other opportunistic pathogens as determined by bacterial culture; and (d) met the Chinese clinical practice guideline for acute infectious diarrhea in children [14]. Exclusion criteria were as follows: (a) aged ≥ 5 years or illness duration ≥ 2 weeks; (b) evidence of infectious diarrhea caused by other pathogens, including bacteria (*Salmonella*, *Shigella*, *Staphylococcus aureus*, and *Escherichia coli*), fungi, parasites (roundworms, hookworms, pinworms), or other enteroviruses; and (c) history of epilepsy or presence of organic neurological lesions, autoimmune diseases, hematological disorders, inflammatory bowel disease, and food protein allergy.

#### 2.1.2. Study Setting and Grouping

This was a retrospective case–control study in which patients were categorized into two groups: NoVE with convulsions group and NoVE without convulsions group (designated as NoVE group). Pediatric patients with confirmed NoVE but without convulsions were included in the NoVE group. For each participant, the following data were collected: (i) demographic characteristics: gender, race, and age; (ii) perinatal factors: birth weight, preterm status, mode of delivery (vaginal delivery or cesarean section), history of neonatal resuscitation, allergy, and rotavirus (RV) vaccination; (iii) clinical features: convulsive history, frequency of diarrhea and vomiting, fever, and dehydration; and (iv) laboratory parameters: routine hematology, serum biochemistry, inflammatory indices, and immune indices.

#### 2.1.3. Screening for Risk Factors Associated With Convulsions

Comparisons between the two groups were first performed for demographic, perinatal, clinical, and laboratory variables. For biochemical analyses, 2 mL of fasting venous blood was collected, and serum was separated by centrifugation at 3500 r/min for 10 min. Levels of aspartate aminotransferase (AST), alanine aminotransferase (ALT), blood urine nitrogen (BUN), creatinine (Cr), creatine kinase (CK), CK‐MB, lactate dehydrogenase (LDH), alpha‐hydroxybutyrate dehydrogenase (α‐HBDH), and electrolytes were measured using an Olympus AU2700 analyzer. Routine hematology tests were performed on 2 mL of blood collected in EDTA‐2K tubes using a Sysmex XE5000 system. Variables with *p* < 0.05 in univariate logistic regression were entered into a multivariate logistic regression model to identify independent risk factors for convulsions. Model performance was assessed using receiver operating characteristic (ROC) curve analysis.

### 2.2. Part II

#### 2.2.1. Reverse Transcription PCR

To determine the VP1 genotype of NoV, stool samples were collected from both the NoVE with convulsions and NoVE without convulsions groups. Solid stool (5–10 g) or watery stool (5–10 mL) was obtained from each subject. Virus RNA was extracted using the commercial E.Z.N.A Viral RNA kit (Cat. No. R6874‐02, Omega Bio‐tek), following the manufacturer’s instructions. Reverse transcription was performed with the PrimeScript RT Reagent Kit. PCR amplification targeted the VP1 region using specific primers for GI and GII genogroups: *GI-SKF*, 5′‐CTGCCCGAATTYGTAAATGA‐3′; *GI-SKR*, 5′‐CCAACCCARCCATTRTACA‐3′; *GII-SKF*, 5′‐CARGARBCNATGTTYAGRTGGATGAG‐3′; and *GII-SKR*, 5′‐CCRCCNGCATRHCCRTTRTACAT‐3′. Amplification conditions were as follows: 98°C for 2 min, followed by 35 cycles of 98°C for 10 s, 55°C for 15 s, and 72°C for 15 s.

#### 2.2.2. Sanger Sequencing and Phylogenetic Analysis

Amplified PCR products were purified and sequenced by Tsingke Biotech (Kunming, China). Genogroup and genotype assignments were performed using the Norovirus Genotyping Tool Version 2.0 (https://www.rivm.nl/mpf/norovirus/typingtool). Sequences were further compared with the reference strains using BLAST (https://www.ncbi.nlm.nih.gov/blast). Representative NoV sequences of different genotypes were retrieved from GenBank. Multiple sequence alignments were generated with ClustalW software. Phylogenetic trees were constructed in MEGA X using the neighbor‐joining method with 1000 bootstrap replicates, as previously described [15]. Detection rates and VP1 positivity were calculated for both groups.

### 2.3. Statistical Analysis

Categorical variables were expressed as counts and percentages, while normally distributed continuous variables were presented as mean ± standard deviation (SD) and compared using Student’s *t*‐test. Skewed data were expressed as median (interquartile range) and analyzed using the Mann–Whitney *U* test. Group comparisons for categorical variables were performed using the chi‐square test. Logistic regression was used to identify risk factors associated with convulsions in NoVE patients, with variables selected using the forced‐entry method. All analyses were conducted with SPSS Version 26.0 (IBM Corp., Armonk, NY, USA). A two‐sided *p* value < 0.05 was considered statistically significant.

## 3. Results

### 3.1. Comparison of Demographic, Birth‐Related, Clinical, and Laboratory Characteristics

In total, 50 children were included in the NoVE with convulsion group, and 45 in the NoVE without convulsion group. All cases met the diagnostic criteria for acute infectious diarrhea, presenting with altered stool characteristics and/or increased stool frequency. There were no significant differences between groups in demographic characteristics (gender, race, allergy history, or RV vaccination) or birth‐related factors (preterm birth, delivery mode, or neonatal resuscitation) (all *p* > 0.05, Table 1). In terms of clinical features, fever and diarrhea frequency were significantly higher in the NoVE with convulsions group compared with the NoVE without convulsions group (all *p* < 0.001). No significant differences were observed in dehydration or vomiting frequency (*p* = 0.375). For the laboratory features, significant differences were detected between groups in BUN, CK, LDH‐MB, CK‐MB, Na^+^, Mg^2+^, and CD3 between the two groups (all *p* < 0.05).

**Table 1:** Comparison of demographic information, birth‐related information, clinical features, and laboratory features between NoVE combined with convulsion and NoVE groups.

Variable	NoVE combined with convulsion group (*n *= 50)	NoVE group (*n *= 45)	*χ* ^2^ value/*z* value	*p* value
Gender			1.799	0.180
Male	22 (44.0%)	26 (57.8%)		
Female	28 (56.0%)	19 (42.2%)		
Age			9.021	0.011
< 12 months	4 (8.0%)	12 (26.7%)		
12–36 months	45 (90.0%)	29 (64.4%)		
> 36 months	1 (2.0%)	4 (8.9%)		
Allergy			2.887	0.089
Yes	4 (8.0%)	9 (20.0%)		
No	46 (92.0%)	36 (80.0%)		
RV vaccination			1.090	0.297
Vaccinated	7 (14.0%)	10 (22.2%)		
Nonvaccinated	43 (86.0%)	35 (77.8%)		
Birth weight			0.018	0.892
Low birth weight	6 (12.0%)	5 (11.1%)		
Normal weight	44 (88.0%)	40 (88.9%)		
Preterm birth			0.038	0.845
Yes	6 (12.0%)	6 (13.3%)		
No	44 (88.0%)	39 (86.7%)		
Type of delivery			0.007	0.931
Vaginal delivery	26 (52.0%)	23 (51.1%)		
Cesarean section	24 (48.0%)	22 (48.9%)		
Rescue			< 0.001	1.000
Yes	10 (20.0%)	9 (20.0%)		
No	40 (80.0%)	36 (80.0%)		
Fever			17.504	< 0.001
Yes	39 (78.0%)	16 (35.6%)		
No	11 (22.0%)	29 (64.4%)		
Dehydration			0.786	0.375
Yes	6 (12.0%)	3 (6.7%)		
No	44 (88.0%)	42 (93.3%)		
Diarrhea	4.00 (3.00, 6.00)	6.00 (3.00, 10.00)	−2.597	0.009
Vomiting	4.00 (2.75, 7.00)	6.00 (3.00, 10.00)	−1.643	0.100
ALT	23.00 (18.50, 28.50)	23.00 (17.00, 41.00)	−0.023	0.982
AST	48.00 (41.00, 58.50)	47.00 (37.50, 57.50)	−0.470	0.639
BUN	4.42 (2.37, 5.84)	2.91 (1.70, 4.56)	−2.258	0.024
Cr	21.27 (18.29, 24.78)	20.85 (18.00, 24.97)	−0.481	0.630
CK	96.00 (62.75, 187.75)	57.00 (38.50, 97.00)	−3.675	< 0.001
CK‐MB	35.00 (26.75, 44.00)	27.00 (20.00, 37.50)	−2.726	0.006
LDH	258.50 (226.75, 302.00)	275.00 (240.00, 337.00)	−1.469	0.142
LDH‐MB	67.50 (60.75, 78.00)	61.00 (56.00, 74.00)	−2.114	0.034
α‐HBDH	199.00 (177.75, 227.25)	201.00 (177.00, 241.50)	−0.037	0.970
Na^+^	136.85 (135.25, 138.00)	138.00 (137.00, 140.10)	−3.209	0.001
K^+^	4.35 ± 0.44	4.43 ± 0.63	−0.776	0.440
Ca^2+^	2.49 ± 0.13	2.44 ± 0.13	1.605	0.112
Mg^2+^	0.80 (0.75, 0.86)	0.88 (0.80, 0.92)	−3.552	< 0.001
Glu	4.00 (3.63, 4.52)	3.90 (3.60, 4.40)	−0.567	0.571
WBC	7.07 (5.62, 8.80)	7.32 (6.12, 12.50)	−1.483	0.138
CRP	1.01 (0.50, 5.50)	0.54 (0.50, 2.38)	−1.020	0.308
PCT	0.24 (0.24, 0.24)	0.24 (0.24, 0.28)	−0.868	0.385
ESR	2.00 (2.00, 3.00)	2.00 (2.00, 6.00)	−2.169	0.030
IgG	5.05 ± 1.18	4.72 ± 1.55	1.117	0.267
IgA	0.29 (0.17, 0.47)	0.37 (0.26, 0.66)	−1.932	0.053
CD3	66.44 ± 6.90	62.05 ± 9.30	2.064	0.044
CD4/CD8	2.35 (1.76, 2.84)	2.05 (1.68, 2.91)	−1.181	0.238

*Note:* NoVE, norovirus enteritis; RV, rotavirus; ALT, alanine aminotransferase; AST, aspartate aminotransferase; Cr, creatinine; LDH, lactate dehydrogenase; α‐HBDH, alpha‐hydroxybutyrate dehydrogenase; Glu, glucose.

Abbreviations: BUN, blood urine nitrogen; CK, creatine kinase; CRP, C‐reactive protein; ESR, erythrocyte sedimentation rate; WBC, white blood cell.

### 3.2. Independent Risk Factors for NoVE With Convulsions

**Figure 1:**
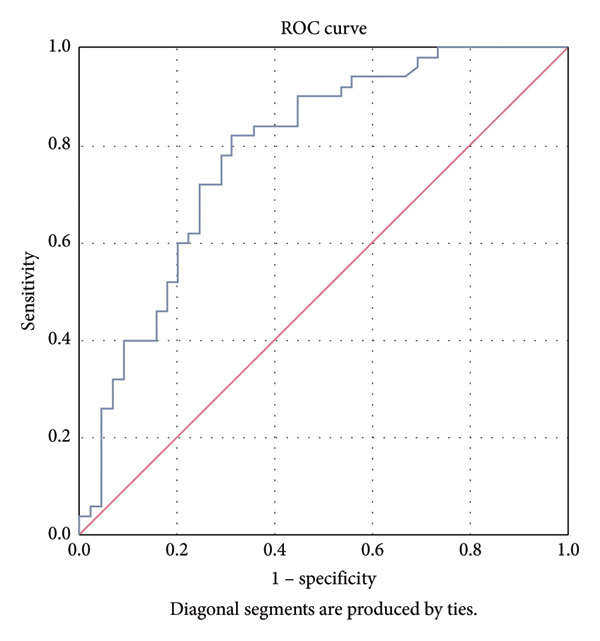
ROC curve for predicting model of the NoVE complicated with convulsions. The generated AUC was 0.787.

Logistic regression analysis identified that elevated serum CK‐MB and fever were independent risk factors for NoVE complicated by convulsions, while lower diarrhea frequency was a protective factor for convulsions in NoVE (Table 2). The simplified logistic regression formula was Logit(P) = 0.497 × CK‐MB‐1.71 × fever‐0.123 × diarrhea frequency. ROC analysis demonstrated good predictive performance of the model for NoVE with convulsions (AUC = 0.787, 95% CI = 0.694, 0.880; *p* < 0.001; Figure 1).

**Table 2:** Multivariate logistic regression analysis for the NoVE complicated with convulsions.

Variables	*β* value	Standard error	Wald *χ* ^2^ value	*p* value	OR value	95% CI
CK‐MB	0.075	0.033	5.205	0.023	1.077	1.011–1.149
Fever	3.567	1.225	8.478	0.004	35.415	3.209–390.846
Less diarrhea frequency	−0.602	0.237	6.481	0.011	0.548	0.344–0.871

*Note:* NoVE, norovirus enteritis; LDH, lactate dehydrogenase.

Abbreviations: BUN, blood urine nitrogen; CI, confidence interval; CK, creatine kinase.

### 3.3. VP1 Genotypes in Both Groups

A total of 157 stool samples were collected from the NoVE and NoVE with convulsion groups. Of these, 114 samples (72.61%) tested positive for NoV, including 81 from the NoVE group and 33 samples from NoVE with convulsion group. Two VP1 genotypes were identified: GII.3 (17 samples) and GII.4 (97 samples). The GII.3 genotype was detected exclusively in 12 NoVE samples (14.81%) and 5 (15.19%) NoVE with convulsion group samples. The GII.4 genotype was present in 28 (84.85%) samples of the NoVE with convulsion group and 69 (85.19%) samples from NoVE group (Table 3).

**Table 3:** Distribution of genotype in the samples.

Genotype	NoVE samples (*n* = 81)	NoVE + convulsion samples (*n* = 33)	*p* value
GII.3	12 (14.81%)	5 (15.19%)	1.00
GII.4	69 (85.19%)	28 (84.85%)	

*Note:* NoVE, norovirus enteritis; the test was performed using the Fisher exact test.

### 3.4. Phylogenetic Analysis

**Figure 2:**
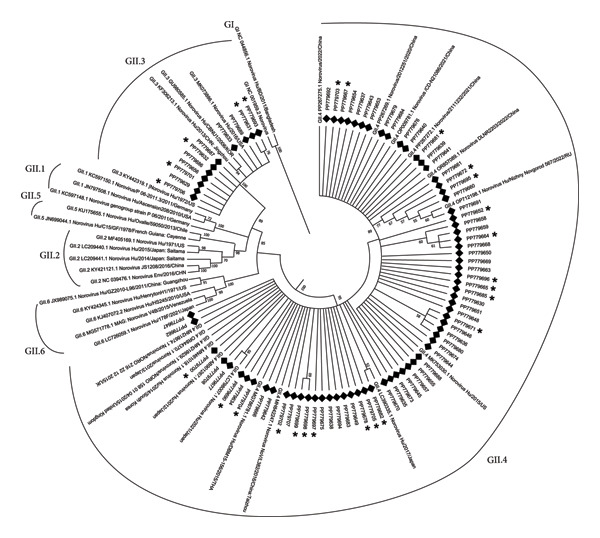
Phylogenetic tree analysis of VP1. The evolutionary tree was constructed using the neighbor‐joining method with 1000 bootstrap replicates in MEGA X. Evolutionary tree analysis showed that 11 of the 80 sequences had a genotype of GII.3, and 69 sequences were of GII.4. Bootstrap values ≥ 50% are shown; values < 70% indicate moderate support.

After removing duplicate sequences, 80 unique sequences were obtained and submitted to GenBank database (Accession No. PP779629‐PP779708). Phylogenetic analysis revealed that 11 sequences belonged to genotype GII.3 and 69 sequences to GII.4 (Figure 2). The patient sequences complicated by convulsions were 5 GII.3 and 28 GII.4. Among the 80 sequences, 69 (86.25%) clustered with GII.4 strains, while 11 sequences (13.75%) clustered with GII.3 strains (Figure 2).

## 4. Discussion

NoV is recognized as a leading cause of acute gastroenteritis in children worldwide, responsible for considerable morbidity due to dehydration, electrolyte imbalance, and acid–base disturbances [16–18]. Beyond gastrointestinal manifestations, an increasing number of studies have reported neurological complications, most notably convulsions, in pediatric patients with NoV infection [19, 20]. Indeed, convulsion is caused by the transient synchronized discharge of a large number of neurons in the brain, resulting in uncontrollable twitching or changes in muscle tone of the voluntary muscles involved and even epilepsy [21, 22]. Frequent and prolonged convulsive attacks can cause nervous system injury in children, thereby affecting the growth and development. At present, the pathogenesis of NoVE complicated by convulsions is unclear as the reasons are complex. Some scholars believe that complicated convulsions are related to infection, high fever, young age, and genetic factors [6]. In this retrospective case–control study, we identified key clinical and laboratory predictors, including elevated CK‐MB levels, fever, and frequency of diarrhea, as independent influencing factors for NoVE complicated with convulsions. Furthermore, genotyping revealed that the majority of strains associated with convulsions belonged to the GII.4 genotype in the NoVE with convulsion group, underscoring its potential role in the pathogenesis of NoV‐related neurological complications.

Previous studies have primarily focused on identifying risk factors for convulsions in children with mild NoVE, such as RV infection, first seizure episode, and a history of convulsions [6, 23, 24]. However, limited research has addressed the specific risk factors associated with NoVE complicated by convulsions. In this study, NoV infection complicated with convulsions was used as the dependent variable, while age, BUN, CK, CK‐MB, LDH‐MB, Na^+^, Mg^2+^, CD3, fever, and diarrhea frequency were included as independent variables in the regression model. Multivariate logistic regression analysis revealed that elevated serum CK‐MB levels and fever were independent predictors of convulsions in NoVE, and lower frequency of diarrhea was a protective factor for convulsions in NoVE. Among these, CK‐MB is a well‐recognized marker of myocardial ischemia and injury [25]. Our findings demonstrated that children with NoVE complicated by convulsions had significantly higher CK‐MB concentrations compared with those without convulsions, suggesting that myocardial involvement may contribute to convulsive episodes. Additionally, the serum sodium Na + concentration in the NoVE with convulsion group was significantly lower than that in the NoVE group. Previous studies have indicated that hyponatremia reduces extracellular osmotic pressure, leading to fluid shifts in brain cells, cerebral edema, and subsequent convulsions. Furthermore, convulsions cause hypoxia and sodium pump dysfunction, which promote intracellular sodium influx and further decrease serum sodium concentrations [26, 27]. In our study, multivariate logistic regression identified that fever was an independent risk factor of convulsions after adjusting for CK‐MB level and diarrhea frequency. This suggests that while fever is common among all NoVE patients, within the broader context of other biological markers, the fever may distinguish those who are at higher risk for convulsions.

NoV infection combined with convulsions is associated with genomic variation. Previous studies have indicated that GII.4 genotype infection was an independent factor associated with convulsions [11, 28]. Globally, GII is the predominant genotype of NoV, particularly the GII.4 genogroup that has caused the majority of outbreaks and sporadic cases [29, 30]. In this study, 2 genotypes (GII.3 and GII.4) were isolated from the 114 stool samples. In the NoVE with convulsion group, GII.4 genotype was the major genotype. This may indicate that GII.4 genotype may associate with the onset of NoVE complicated with convulsion. Previous studies have occasionally detected NoV RNA in the bloodstream (viremia) and, rarely, in cerebrospinal fluid, suggesting the theoretical possibility of CNS involvement [31, 32]. Indeed, GII.4 was the predominant strain with a vast population susceptibility compared with GII.3 genotype. Besides a high similarity with some sequences in China in 2020–2022, the GII.4 sequences were also similar to the 2015 American strain (MK753030), the 2012, 2013, and 2017 Japanese strains (AB901267, OR844370, and LC390335), the 2014 South Korean strain (MN461018), and the 2015 UK strain (MH218629 and MH218674), presenting a high degree of similarity [33–35]. GII.3 sequences were closely related to strains from China (FK306213), USA (KY442319 and MK073886), and South Korea (GU980585) [36–38]. Recent molecular epidemiological studies from South Asia further support our findings. In a birth cohort in Dhaka, Bangladesh (2010–2013), a wide range of GI and GII VP1 genotypes were detected, with GII.4 constituting approximately 20% and GII.3 being among the frequent strains in pediatric gastroenteritis cases [39]. In Pakistan, a hospital‐based study reported a 16.1% prevalence of NoV among children under 5 years of age, with GII genogroup accounting for 73% of infections; both GII.3 and GII.4 were detected, though GII.4 proportions varied by setting [40]. Moreover, a systematic review across Asia highlighted the sustained dominance of GII.4 in India, Bangladesh, Taiwan, and Vietnam, while GII.3 remained more common in Malaysia, Russia, and Bangladesh [41]. These data align with our results, reinforcing the global epidemiological prominence of GII.4 strains and underscoring its potential role in NoVE‐associated convulsions.

Our study has certain limitations. The number of samples is small and it cannot cover all NoV genotypes. Besides, we still cannot identify the potential mechanisms of NoVE complicated with convulsions. We cannot perform the severity stratification (e.g., SCENARIO scores). In the future, more studies along with validation based on severity scores are required to validate our results.

## 5. Conclusions

Our findings indicate that elevated serum CK‐MB levels, fever, and frequency of diarrhea are independent influencing factors for NoVE complicated by convulsions. Moreover, genotyping revealed that the majority of strains associated with convulsions belonged to the GII.4 genotype, underscoring its potential role in the pathogenesis of NoV‐related neurological complications.

## Ethics Statement

The study protocols were in accordance with the Declaration of Helsinki and approved by the Ethics Committee of Kunming Children’s Hospital (approval no. 2022‐03‐009‐K01).

## Consent

Written informed consent was obtained from the guardians of each child.

## Conflicts of Interest

The authors declare no conflicts of interest.

## Author Contributions

Suqi Xu: writing–original draft, validation, resources, methodology, and investigation. Mingying Wang: writing–original draft, data curation, visualization, and formal analysis. Canchun Zhao: methodology, data curation, and investigation. Jiahui Fang: methodology, investigation, and validation. Shumei He: software and investigation. Zhuoheng Li: methodology and visualization. Haoyu Ma: resources and data curation. Qiangming Sun: writing–review and editing, supervision, project administration, and conceptualization. Hongchao Jiang: writing–review and editing, supervision, project administration, and conceptualization. Suqi Xu and Mingying Wang contributed equally to this work.

## Funding

This work was supported by the Xingdian Talent Support Program for Medical and Health Talents Project (XDYC‐YLWS‐2023‐0004; grant to Hongchao Jiang), Kunming Research Center for Pathogen Identification and Pathogenic Mechanism for Emerging and Unknown Infectious Diseases in Children (2023‐SW‐Research‐02; grant to Hongchao Jiang), Kunming Science and Technology Plan Project (2024‐1‐NS‐17; grant to Suqi Xu), and Health Research Project of Kunming Health Commission (2022‐06‐01‐015; grant to Suqi Xu).

## Data Availability

The data generated in this study are available upon request from the corresponding authors.

## References

[1] Robilotti E. , Deresinski S. , and Pinsky B. A. , Norovirus, Clinical Microbiology Reviews. (2015) 28, no. 1, 134–164, 10.1128/cmr.00075-14, 2-s2.0-84920938944. 25567225 PMC4284304

[2] Shah M. P. and Hall A. J. , Norovirus Illnesses in Children and Adolescents, Infectious Disease Clinics of North America. (2018) 32, no. 1, 103–118, 10.1016/j.idc.2017.11.004, 2-s2.0-85041372766. 29406972 PMC6814392

[3] Hasing M. E. , Lee B. E. , Qiu Y. et al., Changes in Norovirus Genotype Diversity in Gastroenteritis Outbreaks in Alberta, Canada: 2012–2018, BMC Infectious Diseases. (2019) 19, no. 1, 10.1186/s12879-019-3792-y, 2-s2.0-85061855288. PMC6381812 30782126

[4] Diao X. , Guo C. , and Li S. , Identification of a Novel Anoikis-Related Gene Signature to Predict Prognosis and Tumor Microenvironment in Lung Adenocarcinoma, Thoracic Cancer. (2023) 14, no. 3, 320–330, 10.1111/1759-7714.14766. 36507553 PMC9870742

[5] Haddadin Z. , Batarseh E. , Hamdan L. et al., Characteristics of GII.4 Norovirus Versus Other Genotypes in Sporadic Pediatric Infections in Davidson County, Tennessee, USA, Clinical Infectious Diseases. (2021) 73, no. 7, e1525–e1531, 10.1093/cid/ciaa1001. 32667045 PMC8492161

[6] Kim G. H. , Byeon J. H. , Lee D. Y. , Jeong H. J. , and Eun B. L. , Norovirus in Benign Convulsions With Mild Gastroenteritis, Italian Journal of Pediatrics. (2016) 42, no. 1, 10.1186/s13052-016-0303-2, 2-s2.0-85009085841. PMC5096325 27809881

[7] Hu M. H. , Lin K. L. , Wu C. T. , Chen S. Y. , and Huang G. S. , Clinical Characteristics and Risk Factors for Seizures Associated With Norovirus Gastroenteritis in Childhood, Journal of Child Neurology. (2017) 32, no. 9, 810–814, 10.1177/0883073817707302, 2-s2.0-85025065776. 28482763

[8] Fang Y. , Dong Z. , Liu Y. et al., Molecular Epidemiology and Genetic Diversity of Norovirus Among Hospitalized Children With Acute Gastroenteritis in Tianjin, China, 2018–2020, BMC Infectious Diseases. (2021) 21, no. 1, 10.1186/s12879-021-06375-2. PMC8277986 34261441

[9] Kowalzik F. , Binder H. , Zöller D. et al., Norovirus Gastroenteritis Among Hospitalized Patients, Germany, 2007–2012, Emerging Infectious Diseases. (2018) 24, no. 11, 2021–2028, 10.3201/eid2411.170820, 2-s2.0-85055079574. 30334712 PMC6199990

[10] Ji L. , Hu G. , Xu D. , Wu X. , Fu Y. , and Chen L. , Molecular Epidemiology and Changes in Genotype Diversity of Norovirus Infections in Acute Gastroenteritis Patients in Huzhou, China, 2018, Journal of Medical Virology. (2020) 92, no. 12, 3173–3178, 10.1002/jmv.26247. 32603477 PMC7692952

[11] Chen Y. F. E. , Wang C. Y. , Chiu C. H. , Kong S. S. , Chang Y. J. , and Chen S. Y. , Molecular Epidemiology and Clinical Characteristics of Norovirus Gastroenteritis With Seizures in Children in Taiwan, 2006–2015, Medicine (Baltimore). (2019) 98, no. 40, 10.1097/md.0000000000017269, 2-s2.0-85072914146. PMC6783164 31577718

[12] Tan M. , Norovirus Vaccines: Current Clinical Development and Challenges, Pathogens. (2021) 10, no. 12, 10.3390/pathogens10121641. PMC8709042 34959596

[13] Plantenga M. S. , Shiferaw B. , Keene W. E. et al., Specimen Collection and Confirmation of Norovirus Outbreaks, Emerging Infectious Diseases. (2011) 17, no. 8, 1553–1555, 10.3201/eid1708.101815, 2-s2.0-79960876939. 21801649 PMC3381577

[14] Chen J. , Wan C. M. , Gong S. T. et al., Chinese Clinical Practice Guidelines for Acute Infectious Diarrhea in Children, World Journal of Pediatrics. (2018) 14, no. 5, 429–436, 10.1007/s12519-018-0190-2, 2-s2.0-85054307166. 30269306

[15] John J. L. , Mori D. , Amit L. N. , Mosiun A. K. , Chin A. Z. , and Ahmed K. , High Proportion of Norovirus Infection and Predominance of GII.3 [P12] Genotype Among the Children Younger Than 5 in Sabah, Malaysian Borneo, Journal of Clinical Virology. (2021) 143, 10.1016/j.jcv.2021.104968. 34509928

[16] Crawford S. E. , Ramani S. , Tate J. E. et al., Rotavirus Infection, Nature Reviews Disease Primers. (2017) 3, no. 1, 10.1038/nrdp.2017.83, 2-s2.0-85033432420. PMC5858916 29119972

[17] Shah G. S. , Das B. K. , Kumar S. , Singh M. K. , and Bhandari G. P. , Acid Base and Electrolyte Disturbance in Diarrhoea, Kathmandu University Medical Journal. (2007) 5, no. 1, 60–62. 18603987

[18] Ahmad M. S. , Wahid A. , Ahmad M. , Mahboob N. , and Mehmood R. , Prevalence of Electrolyte Disorders Among Cases of Diarrhea With Severe Dehydration and Correlation of Electrolyte Levels With Age of the Patients, Journal of College of Physicians and Surgeons Pakistan. (2016) 26, no. 5, 394–398. 27225145

[19] Shi K. , Jiang D. , Yang J. , Li Y. , Chen W. , and Li P. , Clinical Characteristics and Follow-Up of Children With Norovirus-Associated Benign Convulsions With Mild Gastroenteritis, Epilepsia Open. (2023) 8, no. 3, 1049–1053, 10.1002/epi4.12782. 37394877 PMC10472357

[20] Lu M. C. , Lin S. C. , Hsu Y. H. , and Chen S. Y. , Epidemiology, Clinical Features, and Unusual Complications of Norovirus Infection in Taiwan: What We Know After Rotavirus Vaccines, Pathogens. (2022) 11, no. 4, 10.3390/pathogens11040451. PMC9026459 35456126

[21] Sacktor B. , Wilson J. E. , and Tiekert C. G. , Regulation of Glycolysis in Brain, in Situ, During Convulsions, Journal of Biological Chemistry. (1966) 241, no. 21, 5071–5075, 10.1016/s0021-9258(18)99671-7. 4380842

[22] Yang H. , Wu J. , Guo R. et al., Glycolysis in Energy Metabolism During Seizures, Neural Regeneration Research. (2013) 8, no. 14, 1316–1326, 10.4103/1673-5374.121652. 25206426 PMC4107649

[23] Shi K. , Yang J. , Wu Y. , Han H. , Guo J. , and Chen W. , Risk Factors for the Recurrence of Convulsions With Mild Gastroenteritis in Children, Seizure. (2020) 80, 192–195, 10.1016/j.seizure.2020.06.016. 32619828

[24] Ogawa C. , Kidokoro H. , Ishihara N. et al., Splenial Lesions in Benign Convulsions With Gastroenteritis Associated With Rotavirus Infection, Pediatric Neurology. (2020) 109, 79–84, 10.1016/j.pediatrneurol.2019.05.002. 32303390

[25] Sax H. , Contesse J. , Dubach P. , and Reinhart W. H. , Creatine Kinase MB During Myocardial Infarction: Relationship to Preexisting Coronary Heart Disease and Medication, Acta Cardiologica. (1997) 52, no. 5, 423–430. 9428940

[26] Fang C. , Fan W. , Zhang C. , and Yang Y. , Risk Factors for Benign Convulsions With Mild Gastroenteritis, Frontiers in Pediatrics. (2022) 10, 10.3389/fped.2022.925896. PMC9277103 35844760

[27] Higuchi Y. , Kubo T. , Mitsuhashi T. et al., Clinical Epidemiology and Treatment of Febrile and Afebrile Convulsions With Mild Gastroenteritis: A Multicenter Study, Pediatric Neurology. (2017) 67, 78–84, 10.1016/j.pediatrneurol.2016.05.011, 2-s2.0-85009723992. 28094168

[28] Tsai C. N. , Lin C. Y. , Lin C. W. , Shih K. C. , Chiu C. H. , and Chen S. Y. , Clinical Relevance and Genotypes of Circulating Noroviruses in Northern Taiwan, 2006–2011, Journal of Medical Virology. (2014) 86, no. 2, 335–346, 10.1002/jmv.23728, 2-s2.0-84890192517. 24009100

[29] Chan M. C. , Lee N. , Hung T. N. et al., Rapid Emergence and Predominance of a Broadly Recognizing and Fast-Evolving Norovirus GII.17 Variant in Late 2014, Nature Communications. (2015) 6, no. 1, 10.1038/ncomms10061, 2-s2.0-84949034565. PMC4686777 26625712

[30] Ai J. , Zhang M. , Jin F. , and Xie Z. , Recombinant GII.4[P31] was Predominant Norovirus Circulating in Beijing Area, China, 2018–2020, Virologica Sinica. (2021) 36, no. 5, 1245–1247, 10.1007/s12250-021-00381-z. 33835390 PMC8034047

[31] Deb S. , Mondal R. , Lahiri D. et al., Norovirus-Associated Neurological Manifestations: Summarizing the Evidence, Journal of NeuroVirology. (2023) 29, no. 4, 492–506, 10.1007/s13365-023-01152-0. 37477790 PMC10501950

[32] Lucero Y. , Matson D. O. , Ashkenazi S. , George S. , and O’Ryan M. , Norovirus: Facts and Reflections From Past, Present, and Future, Viruses. (2021) 13, no. 12, 10.3390/v13122399. PMC8707792 34960668

[33] Scully T. , Ettela A. , LeRoith D. , and Gallagher E. J. , Obesity, Type 2 Diabetes, and Cancer Risk, Frontiers in Oncology. (2020) 10, 10.3389/fonc.2020.615375. PMC7884814 33604295

[34] Ushijima H. , Hoque S. A. , Akari Y. et al., Molecular Evolution of GII.P31/GII.4_Sydney_2012 Norovirus Over a Decade in a Clinic in Japan, International Journal of Molecular Sciences. (2024) 25, no. 7, 10.3390/ijms25073619. PMC11011564 38612429

[35] Zhu X. , He Y. , Wei X. et al., Molecular Epidemiological Characteristics of Gastroenteritis Outbreaks Caused by Norovirus GII.4 Sydney [P31] Strains—China, October 2016–December 2020, China CDC Weekly. (2021) 3, no. 53, 1127–1132, 10.46234/ccdcw2021.276. 35036035 PMC8742140

[36] Johnson J. A. , Parra G. I. , Levenson E. A. , and Green K. Y. , A Large Outbreak of Acute Gastroenteritis in Shippensburg, Pennsylvania, 1972 Revisited: Evidence for Common Source Exposure to a Recombinant GII.Pg/GII.3 Norovirus, Epidemiology and Infection. (2017) 145, no. 8, 1591–1596, 10.1017/s0950268817000498, 2-s2.0-85015201350. 28294087 PMC6691898

[37] Fu J. , Ai J. , Bao C. et al., Evolution of the GII.3[P12] Norovirus From 2010–2019 in Jiangsu, China, Gut Pathogens. (2021) 13, no. 1, 10.1186/s13099-021-00430-8. PMC8149921 34039425

[38] Strubbia S. , Schaeffer J. , Besnard A. et al., Metagenomic to Evaluate Norovirus Genomic Diversity in Oysters: Impact on Hexamer Selection and Targeted Capture-Based Enrichment, International Journal of Food Microbiology. (2020) 323, 10.1016/j.ijfoodmicro.2020.108588. 32200157

[39] Nelson M. I. , Mahfuz M. , Chhabra P. et al., Genetic Diversity of Noroviruses Circulating in a Pediatric Cohort in Bangladesh, Journal of Infectious Diseases. (2018) 218, no. 12, 1937–1942, 10.1093/infdis/jiy454, 2-s2.0-85056271888. 30053045 PMC6217719

[40] Alam A. , Qureshi S. A. , Vinjé J. , and Zaidi A. , Genetic Characterization of Norovirus Strains in Hospitalized Children From Pakistan, Journal of Medical Virology. (2016) 88, no. 2, 216–223, 10.1002/jmv.24329, 2-s2.0-84954387903. 26175018 PMC5916762

[41] Singh A. K. , Nagar J. , Tandekar A. et al., The Evolving Landscape of Norovirus GII Genotypes in Asia: A Systematic Review and Meta-Analysis, Journal of Clinical Virology. (2025) 179, 10.1016/j.jcv.2025.105809. 40466412

